# Metastasectomy and Stereotactic Body Radiotherapy for Colorectal Cancer With Liver and Lung Oligometastases: A Case Report of Complete Remission in a 96-Year-Old Patient

**DOI:** 10.7759/cureus.58135

**Published:** 2024-04-12

**Authors:** Ming Pan

**Affiliations:** 1 Radiation Oncology, Windsor Regional Hospital Cancer Program, Windsor, CAN

**Keywords:** complete remission, sabr, stereotatic body radiotherapy, liver metastasectomy, lung oligometastases, colorectal cancer

## Abstract

We report a rare case of an extremely old colorectal cancer (CRC) patient who had complete remission after liver metastasectomy and stereotactic body radiotherapy (SBRT) to lung oligometastases (OM), with good quality of life and no evidence of recurrence 12 years after the initial diagnosis. An 83-year-old male patient had a right hemicolectomy for stage pT3 pN0 adenocarcinoma of the colon. Soon he was found to have liver metastasis treated with radiofrequency ablation and then liver metastasectomy with clear margins, followed by chemotherapy in the form of FOLFIRI for six months. Six years later, positron emission tomography (PET) showed 1.6 cm OM in the left upper lobe lung. He was not considered a good candidate for surgery. We offered him SBRT 48 Gy in four fractions every other day. The lesion disappeared with no recurrence in the same location on PET and serial computed tomography (CT) scans. Three years later, PET-CT found a new OM in the left lingular lung measuring 1.2 cm. A CT-guided lung biopsy confirmed invasive adenocarcinoma favoring OM from the CRC. SBRT planning failed due to its proximity to the heart. He accepted the longer course of conventional volumetric modulated arc therapy at 60 Gy in 15 fractions with daily cone-beam CT guidance. Again, he tolerated treatment very well with no significant side effects, despite his age. He did not require any chemotherapy or other systemic treatment in the last 11 years, so he did not experience any toxicities related to such treatment. This case is important to show that old age alone should not be considered a contraindication for metastasectomy and SBRT for CRC with liver and lung OM.

## Introduction

Stage IV colorectal cancer (CRC) has a poor prognosis, with a five-year overall survival rate of about 14% [1]. The development of new systemic treatments in recent years has moderately improved these patients’ outcomes [2]. They are more likely to suffer symptoms from local diseases due to their extended life expectancy than those diagnosed way before the era of modern chemotherapy and immunotherapy. Radiotherapy has long been considered a palliative treatment for local symptom control with no survival benefit for stage IV CRC patients [3,4]. However, recent studies have shown that the use of radical treatments such as metastasectomy or stereotactic body radiotherapy (SBRT) might achieve excellent local control (LC) and even prolonged survival [5,6]. However, extremely old patients were usually excluded from these studies due to the assumption that they might not be able to tolerate these radical treatments and that they have a much shorter life expectancy. We report the case of a 96-year-old male CRC patient who had complete remission (CR) after liver metastasectomy and SBRT to oligometastases (OM) in the lung, with good quality of life (QoL) and no evidence of recurrence 12 years after the initial diagnosis.

## Case presentation

**Figure 1:**
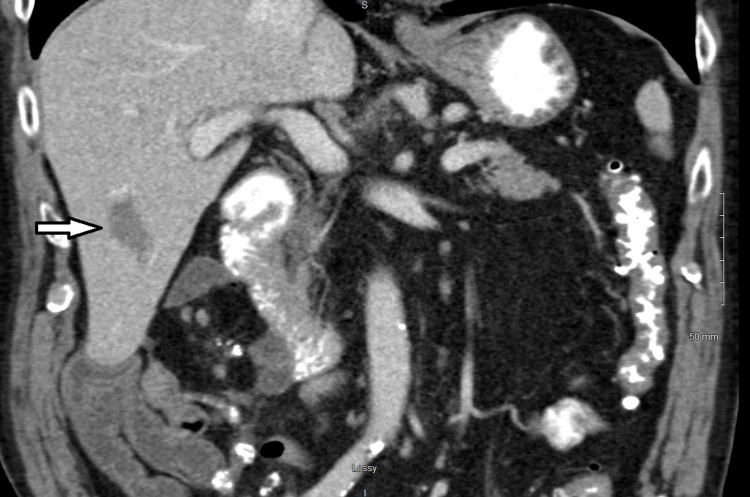
CT after RFA to biopsy-confirmed liver OM (white arrow) in 2012 CT, computed tomography; OM, oligometastases; RFA, radiofrequency ablation

An 83-year-old male patient, previously healthy without any major past medical history (Eastern Cooperative Oncology Group (ECOG) performance status score = 0), had a right hemicolectomy in February 2011 for a stage pT3 pN0 (American Joint Committee on Cancer eighth version) adenocarcinoma of the colon with clear margins and 24 negative lymph nodes. Soon he was found to have a biopsy-proven 2.7 cm liver metastasis in segment V on computed tomography (CT) in December 2011, treated with radiofrequency ablation in January 2012 (Figure 1), and then had a liver metastasectomy with clear margins in March 2012, followed by further chemotherapy in the form of FOLFIRI for six months.

**Figure 2:**
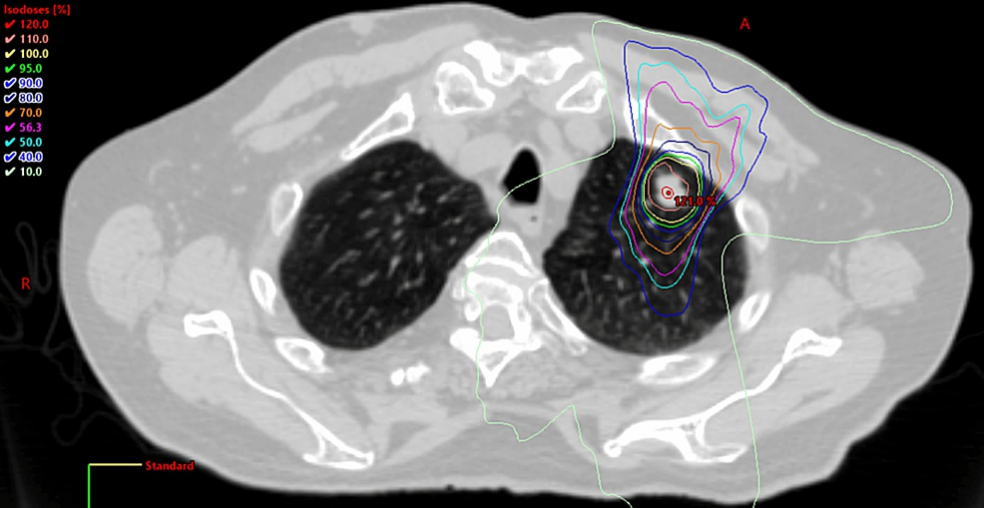
SBRT plan of the first left lung OM in 2018 OM, oligometastases; SBRT, stereotactic body radiotherapy

**Figure 3:**
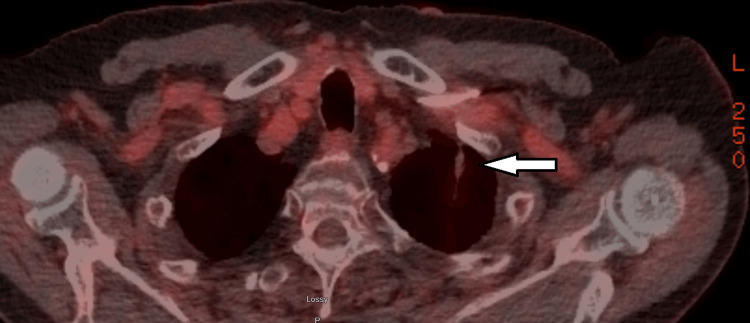
PET showing CR after SBRT to the first lung OM (arrow) CR, complete remission; OM, oligometastases; PET, positron emission tomography; SBRT, stereotactic body radiotherapy

The patient’s serum carcinoembryonic antigen was always normal, with the highest level at 2.8 ng/ml in May 2020. He had no evidence of any recurrent cancer until a slowly growing 1.6 cm left upper lobe lung spiculated nodule was found on CT in December 2017 and was positive on a positron emission tomography (PET) scan with a standardized uptake value (SUV) of 6.6. There were no other suspicious lesions elsewhere. He was not considered a good candidate for any surgery or biopsy due to his age of 90 and the location of his tumor being too close to the major blood vessels. His case was discussed in our tumor board meeting. The consensus was to treat this lesion with SBRT, using the same protocol for stage I non-small cell lung cancer (NSCLC), or OM [7]. He accepted high-dose SBRT at 48 Gy in four fractions every other day in February 2018 (Figure 2), prescribed to the planning target volume (PTV), with 100% of the dose covering 95% of the PTV and 100% of the gross tumor volume (GTV). The lesion disappeared along with some remaining scar tissue on serial CT scans. There has been no recurrence in the same location on the PET scan (Figure 3).

The patient was doing very well during the COVID-19 pandemic. He continued to do his two-mile-in-40-minute walk on a daily basis. However, one day he fell after a syncope in October 2020. He was taken by ambulance to the emergency room. His heart rate dropped to 30 beats per minute. A permanent pacemaker was inserted. While he was in the hospital, he had a chest X-ray and also a CT scan of the head, which did not show any abnormalities. However, a CT scan of the chest and abdomen showed a new nodule in the left lingular lung measuring up to 1.2 cm. A CT-guided lung biopsy was done. The pathology confirmed invasive adenocarcinoma, favoring metastasis from CRC rather than primary lung cancer. The tumor cells were positive for CK20, CDX2, and villin. They were negative for CK7, TTF-1, and napsin A.

**Figure 4:**
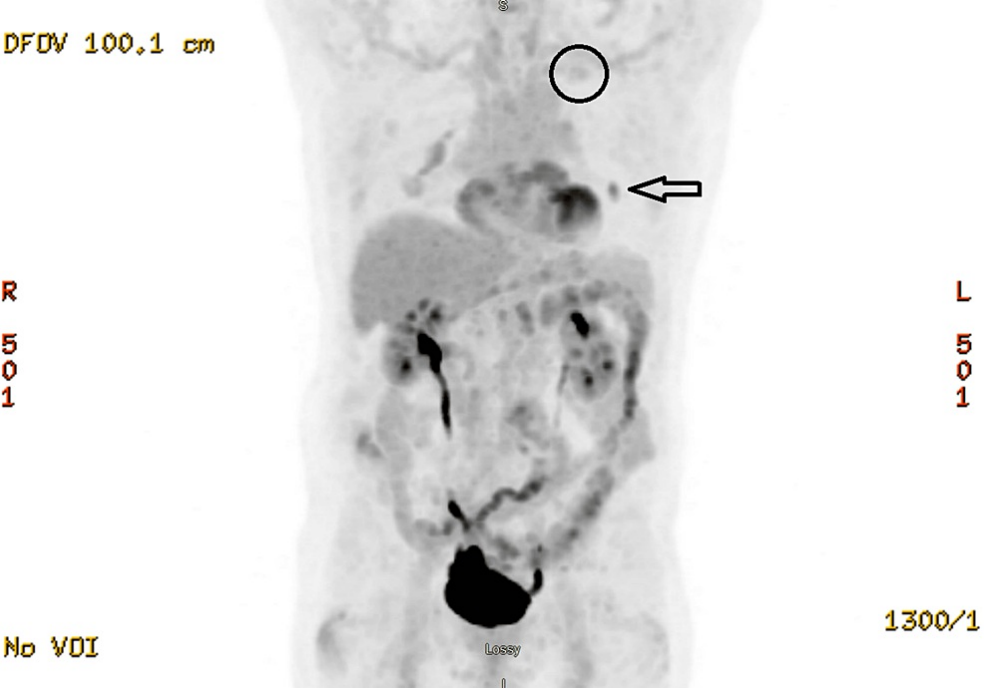
The second biopsy confirmed lung OM on PET in November 2020 (arrow). Note the disappearance of the first lung OM after SBRT in February 2018 (circle) OM, oligometastases; PET, positron emission tomography; SBRT, stereotactic body radiotherapy

His case was discussed in our multidisciplinary cancer conference lung rounds. His colonoscopy was reported to be normal. A PET scan from November 2020 did not show other metastases anywhere in the body. The only positive finding is the 1.2 cm left lingular nodule with SUV 5.1 (Figure 4). There was no abnormal uptake in the first lung OM that received SBRT in 2018. The consensus was to treat the new lung OM with SBRT.

**Figure 5:**
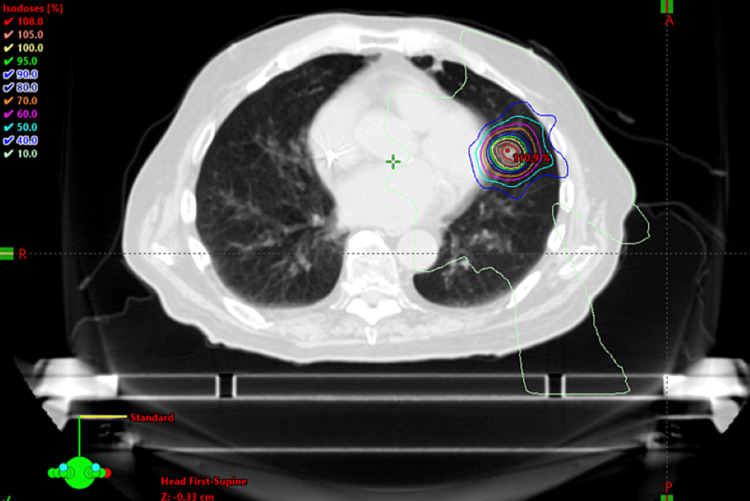
SBRT plan of the second left lung OM in 2020. Note the proximity to the heart. The patient received 60 Gy in 15 fractions with VMAT instead of SBRT OM, oligometastases; SBRT, stereotactic body radiotherapy; VMAT, volumetric modulated arc therapy

The patient underwent another four-dimensional CT simulation for treatment planning purposes. Unfortunately, the GTV was too close to the heart and the pacemaker. We could not pass the organs-at-risk dose constraint criteria on the dose-volume histogram (DVH), so we opted for the longer course of conventional radiotherapy at 60 Gy in 15 fractions with daily cone-beam CT guidance (Figure 5). He completed volumetric modulated arc therapy in December 2020. Again, he tolerated treatment very well with no significant side effects, despite his age. He denied any new respiratory symptoms. He had no concerns about his new pacemaker. CT scans showed CR from conventional hypofractionated radiotherapy.

**Figure 6:**
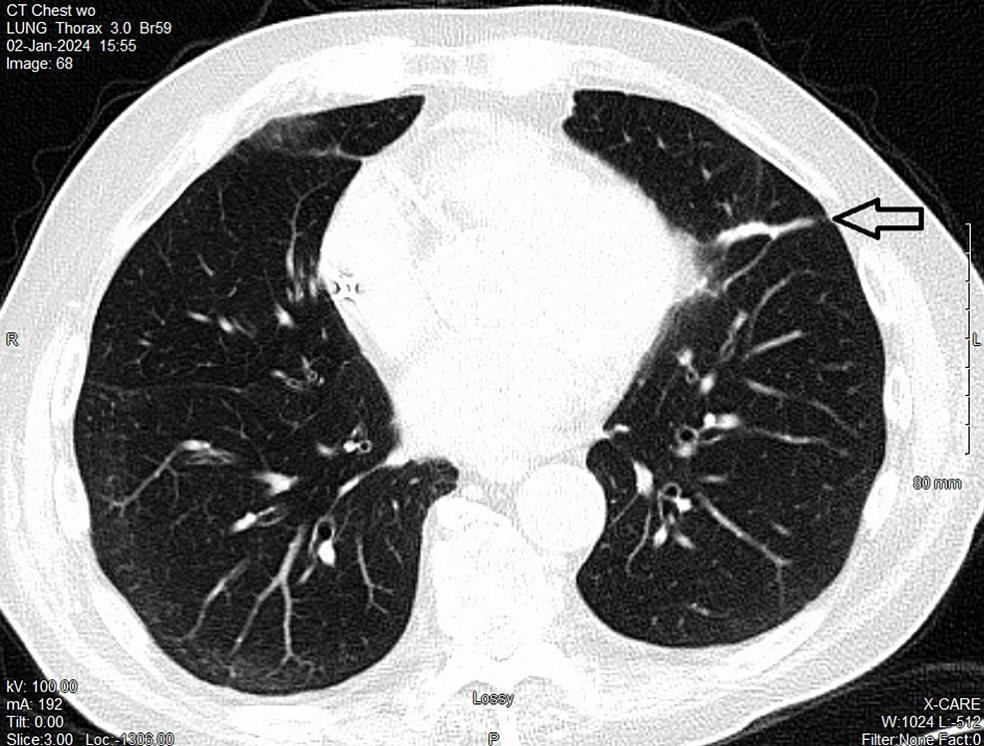
CT in 2024 showing stable scar tissue without recurrence three years after VMAT to the second lung OM CT, computed tomography; OM, oligometastases; VMAT, volumetric modulated arc therapy

Of note, the patient did not receive any chemotherapy or other systemic treatment in the last 11 years, so he did not experience any toxicities related to such treatment. He was walking around without a cane, golfing, and doing daily activities, including gardening, with no difficulties. His ECOG performance status score remained at 0. Unfortunately, he tested positive for COVID-19 in September 2022. This progressed to pneumonia, hypoxemic respiratory failure, and heart failure, so he was admitted to the hospital. He fully recovered and was discharged home two weeks later, having received four doses of the Pfizer COVID-19 vaccination previously. On the last follow-up visit in January 2024, he was stable with no signs of COVID-19. He started to use a walking cane. There was absolutely no evidence of any new primary cancer or metastases on CT (Figure 6). Although he had two courses of high-dose radiation to his left lung, he did survive the COVID-19 pneumonia, suggesting that radiation did not cause any severe damage to his lungs. At the age of 96, he agreed to be followed every six months with a repeat CT scan prior to each visit.

## Discussion

A Canadian national survey found that 85% of general practitioners (GPs) had provided care for patients with advanced cancer within the previous month [8]. It has been known that OM has better outcomes than widespread metastases when treated aggressively [9-11]. Multidisciplinary care, including surgery, radiation, and systemic treatments, in the era of modern chemotherapy and immunotherapy has extended the life expectancy of these patients, especially with metastatic CRC [12,13]. Radiotherapy is no longer just a palliative treatment but also has survival benefits for selected stage IV CRC patients with OM [3,4,13]. Several studies have shown that the use of radical treatments such as metastasectomy or SBRT can improve both LC and OS. They might also improve QoL by delaying systemic treatments that have severe toxicities [2,5,6,13-15].

The Dutch Lung Cancer Audit for Surgery analyzed 1,237 patients after metastasectomy, of whom 127 underwent repeat metastasectomy, for colorectal pulmonary metastases from January 2012 to December 2019. Five-year OS was 53% and 52%, respectively (p = 0.852). More patients suffered from postoperative complications after repeat metastasectomy (18.1% versus 11.6%, respectively; p = 0.033) [14]. Unfortunately, not every patient is a good surgical candidate due to their underlying comorbidities or old age.

For medically inoperable patients or those who refused surgery, it makes more sense to offer them SBRT since its use for early-stage primary NSCLC reported excellent LC [7]. Agolli et al. reported its safety with no grade ≥3 toxicities recorded in the univariate analysis, but long-term LC and OS were much lower than the surgical series [15]. This is especially true when liver OM is included [12]. One reason could be that the optimal SBRT dose for lung OM from CRC is not clear. Wang et al. reported that LC after SBRT from 48 Gy to 60 Gy in four to five fractions was significantly worse than that of primary NSCLC. The one-, three-, and five-year LC were 80.6% vs. 100%, 68.6% vs. 97.2%, and 68.6% vs. 81.0%, respectively. However, their sample size was too small, i.e., only 15 CRC patients were studied, and the CRC group had more multiple lung lesions than the NSCLC group [16]. Another reason could be selection bias, i.e., the patients are usually older and sicker in the SBRT group than in the surgery group. There are always debates between these two options, but Garcia-Exposito et al. reported similar outcomes [17].

Usually, extremely old patients were excluded from these studies due to the assumption that they might not be able to tolerate radical treatments and that they have a much shorter life expectancy. Long-term CR after metastasectomy of liver OM and SBRT to lung OM is rare. These patients were often offered pure palliative dose radiotherapy for a better QoL and no option of radical dose SBRT for cure or longer survival.

Surprisingly, our patient in this case report did so well after high-dose radiation treatment for two lung OM lesions from stage IV colon cancer in 2018 and 2020. The new CT scan did not show any interval changes, progression, or new lung metastases. The remaining bandlike opacity could be scar tissue or fibrosis after high-dose radiation, as it became PET negative. This is consistent with recent publications about SABR to oligo lung OM from CRC with good results [18].

Sheikh et al. reported 381 extra-cranial OM (≤ five lesions) in 235 CRC patients treated with SBRT at six academic cancer centers. The five-year OS and local recurrence rates were 35.9% and 44.3%, respectively. The median OS was 49 months. Biologically effective doses for the α/β ratio of 10 in early-responding tissues and tumor (BED10) of ≥120 Gy, and lung versus liver OM were associated with a lower local recurrence rate [18]. In contrast, the BED10 of the radiation treatment for the two lung OMs of our patient is 105.6 Gy and 84 Gy, respectively. These are much lower than the recommendations in the literature, suggesting other factors, such as the accuracy of GTV contouring and PTV coverage on DVH, should have been considered as confounders. So high-quality prospective trials are still needed to further confirm the role of metastasectomy and SBRT in better LC, prolonged OS, and good QoL, which are scarce in the current literature [19].

Both the patient and his wife were thankful that we did offer him radical dose radiation instead of palliative treatment. They both felt that publishing this case report might benefit other similar CRC patients in the future. There are some educational values in practice. For GPs, is there anything else that you might do differently as a result of reviewing this case report? Think about the last time you cared for an elderly patient with metastatic CRC. How long did you consider they could survive with palliative care? Would you feel more confident referring them to an oncologist for radical dose radiotherapy to help control OM and prolong their survival?

## Conclusions

Our 96-year-old CRC patient had CR after liver metastasectomy and SBRT to lung OM, with good QoL and no evidence of recurrence 12 years after the initial diagnosis. This case is important to show the possibility for extremely old patients with good performance status to tolerate radical treatments for OM and achieve a longer life expectancy. However, high-quality prospective randomized studies are still needed to further clarify the role of metastasectomy and SBRT in stage IV CRC patients with OM.

## Appendices

The patient’s involvement in the creation of this article

The patient and his family had the opportunity to review and comment on the draft manuscript. They did not feel any modifications were necessary and did share their experiences of his treatment.

From the patient’s perspective

I was expecting a short survival time when I was diagnosed with stage IV colon cancer in 2011. I really appreciate that my oncologists offered me surgery and high-dose radiation instead of palliative treatment. I did not have any noticeable discomfort during radiation, and I am excited to celebrate my 96th birthday. I believe the lessons learned from my case could help future cancer patients, especially if they are younger than me.
